# Supplementary material for: Population travel increases the risk of Plasmodium falciparum infection in the highland population of Gardula Zone, South Ethiopia: A longitudinal study
Source: PLoS One. 2024 Dec 20;19(12):e0315900. doi: 10.1371/journal.pone.0315900 (PMC11661618; doi:10.1371/journal.pone.0315900)
Supplement: Attachment — Submitted filename: Response to Reviewers.docx [file pone.0315900.s002.docx]

**Response to Reviewers**

First of all, we would like to extend our thankfulness to Editor’s and reviewers for their detailed appraisal. A response that addresses the points raised during the review process are given below.

Thank you!

**-------------------------//--------------------------**

**A) Academic editor:**

Figure 1 appears to represent Ethiopia and Southern Ethiopia as two separate countries. Please revise the figures accordingly.

**Response:** The comment is well taken and Figure 1 is revised. Accordingly, the caption is also re-narrated (See Figure 1 (separate file), and the caption on page 6 in the manuscript)

Additionally, instead of using the term "illiterate," I recommend using "participants with no formal education," as the translation of "illiterate" into the local language may be considered offensive.:

**Response:** The comment is well taken and amended accordingly (See pages 2 and 22).

**Journal Requirement**

Please review your reference list to ensure that it is complete and correct. If you have cited papers that have been retracted, please include the rationale for doing so in the manuscript text, or remove these references and replace them with relevant current references. Any changes to the reference list should be mentioned in the rebuttal letter that accompanies your revised manuscript. If you need to cite a retracted article, indicate the article’s retracted status in the References list and also include a citation and full reference for the retraction notice.

**Response:** The reference list is reviewed to ensure its completeness and correctness. Accordingly, corrections have been made (see pages 25-30).

**Journal requirement**

The previous submission has no line numbers. Now, the line numbers are inserted in the manuscript file.

Formatting issues: line spacing has been checked and corrected throughout the document

**B) Reviewers:** The reviewers were satisfied with the previous response given. It can be seen from their response.

**C) Other corrections**

Ethiopia has several administrative regions. This study was conducted in former South Nations, Nationalities and People’s Regional States (SNNPRs). Of course, it is political boundary. Currently the region is separated into four administrative regions. The region in which this study was conducted is found in South Ethiopia. To indicate this geographical location the phrase ‘Southwest Ethiopia’ is replaced by ‘South Ethiopia’ in the MS (see pages 1.2, 5,6, Figure Tables).

The study district ‘Dirashe district’ is currently named as ‘Gardula Zone’ with the same geographical location following the division of the former SNNPRs. Still, it is an administrative boundary. To indicate this name change with the same geographical area, the phrase ‘Dirashe district’ is replaced by ‘Gardula Zone’ in the MS (see the Title, Tables and sections of the MS).
